# Case Report: Successful salvage therapy with sulbactam-durlobactam combined with meropenem for carbapenem-resistant Acinetobacter baumannii bloodstream infection in a child after failure of allogeneic hematopoietic stem cell transplantation

**DOI:** 10.3389/fped.2026.1913512

**Published:** 2026-09-10

**Authors:** Qian Zhang, Chunjing Wang, Zhiheng Ding, Jiayi Lyu, Yu Zhang, Fangyuan Lai, Ruihao Zheng, Sixi Liu

**Affiliations:** 1 Department of Hematology and Oncology, Shenzhen Children’s Hospital, Shenzhen, China; 2 Department of Pharmacy, Shenzhen Children’s Hospital, Shenzhen, China

**Keywords:** bloodstream infection, carbapenem-resistant Acinetobacter baumannii, case report, graft failure, hematopoietic stem cells transplantation, pediatrics, sulbactam-durlobactam

## Abstract

Bloodstream infections (BSI) caused by carbapenem-resistant Acinetobacter baumannii (CRAB) constitute a serious complication in patients undergoing hematopoietic stem cell transplantation (HSCT), with pediatric patients facing particularly formidable therapeutic challenges. The phase III ATTACK trial demonstrates that sulbactam-durlobactam (SUL-DUR), a novel β-lactam-β-lactamase inhibitor combination is non-inferior to colistin and is associated with fewer adverse events in adults. However, evidence for SUL-DUR use in post-transplant pediatric patients remains extremely limited. This article reports the first global case of a pediatric patient who experienced two successive HSCT failures and was successfully salvaged with SUL-DUR combined with meropenem for CRAB associated BSI after failing to respond to high-dose sulbactam (150 mg/kg/day) and tigecycline during persistent severe neutropenia, providing a novel strategy for managing resistant bacterial infections in high-risk transplant populations.

## 1 Introduction

Carbapenem-resistant Acinetobacter baumannii (CRAB) has been designated a critical-priority pathogens by the World Health Organization because of its extensively drug-resistance and substantial threat to human health (1). CRAB exhibits resistance to nearly all β-lactam antibiotics, including carbapenems, by producing class D β-lactamases (e.g., OXA-23, OXA-24/40, OXA-58) (2, 3). The mortality rate associated with invasive CRAB infections varies considerably across studies and anatomical sites, with overall reported ranges of approximately 17.7%–57.4% (4, 5), and can be even higher in severe scenarios such as bloodstream infection (BSI), where short-term mortality in some studies approaches or exceeds 50% (6, 7). According to China Antimicrobial Surveillance Network surveillance data, clinical CRAB isolates retain highly susceptible only to polymyxin B and tigecycline. However, both agents are associated with a high risk of hepatorenal toxicity and coagulation disorders, severely limiting their clinical application in hematopoietic stem cell transplantation (HSCT) recipient (8, 9).

Sulbactam-durlobactam (SUL-DUR) is a novel β-lactam-β-lactamase inhibitor combination that exerts synergistic antibacterial activity through a unique dual mechanism (10). Sulbactam directly targets penicillin-binding proteins (PBP1a/b, PBP3) to disrupt bacterial cell wall synthesis, while durlobactam potently inhibits class A/C/D β-lactamases (including OXA-type carbapenemase) to protect sulbactam from hydrolysis (11). SUL-DUR has been approved by the U.S. Food and Drug Administration for the treatment of hospital-acquired bacterial pneumonia and ventilator-associated bacterial pneumonia caused by susceptible CRAB strains in adults (10, 11). Furthermore, the phase III ATTACK trial (NCT03894046) demonstrates that SUL-DUR is non-inferior to colistin in efficacy for CRAB infections and significantly reduces the risk of nephrotoxicity compared to colistin (12). Besides, SUL-DUR achieved favorable pharmacokinetic exposure and therapeutic plasma concentrations in patients with CRAB-associated HABP and VABP, suggesting its potential clinical utility in treating BSI (12).

The usage of SUL-DUR for CRAB BSI has been documented in some adult reports (13). Nevertheless, evidence for SUL-DUR use in the pediatric population remains extremely scarce, with existing literature limited to sporadic case reports (14). Therefore, there is a need for real-world clinical evidence regarding the efficacy and safety of SUL-DUR in pediatric patients. Here, we report the first case of a pediatric patient who developed CRAB associated BSI and successfully treated with sulbactam-durlobactam.

## 2 Case presentation

### 2.1 First HSCT episode

A 10-year-old boy with severe β-thalassemia underwent haploidentical HSCT from his sister on September 22, 2025, and was subsequently diagnosed with graft failure because the donor chimerism was <5% for both myeloid and lymphoid lineages and the absolute neutrophil count did not recover ≥500/μL by day +30 post-transplant with associated pancytopenia. On November 15, 2025 (day +54 post-transplant), he sustained a fall resulting in a subdural hematoma and underwent emergency craniotomy with hematoma evacuation and mechanical ventilation. Postoperative consciousness was regained, and he was successfully weaned from the ventilator. On February 8, 2026 (day +139 post-transplant), the absolute neutrophil count recover ≥500/μL but hemoglobin and platelets still rely on transfusion.

### 2.2 Second HSCT episode

Due to persistent hemoglobin and platelet transfusion dependence, the patient received a second haploidentical HSCT from his father on April 9, 2026. The conditioning regimen consisted of bortezomib (1.3 mg/m^2^ × 4 doses), rituximab (375 mg/m^2^), melphalan (70 mg/m^2^ × 2days), rabbit anti-thymocyte globulin (2 mg/kg × 3days). Graft-vs.-host disease prophylaxis included post-transplant cyclophosphamide (50 mg/kg × 2days), tacrolimus and mycophenolate mofetil.

#### 2.2.1 Regular stool culture screening for CRAB

During the myelosuppression phase following conditioning regimen, the patient developed neutropenia again from April 3. The patient had grade II oral mucositis with herpes simplex virus positive and he was treated with acyclovir. Concurrently, antibacterial and antifungal prophylaxis (days +3 to +8 post-transplant) consisted of cefoperazone-sulbactam (sulbactam dose 80 mg/kg/d) + voriconazole. Regular stool culture screening for carbapenem-resistant organisms on April 10 and April 12 showed CRAB positive and the specific drug susceptibility results are presented in Table 1. As the patient was afebrile and without diarrhea, colonization was considered the most likely explanation, and oral minocycline was added from April 17 for decolonization.

**Table 1:** Antibiotic susceptibility of the Acinetobacter baumannii isolate obtained from stool culture.

Antibiotic	Method	Result (µg/mL)	Interpretive ranges	Interpretation
Meropenem	MIC	≥16	2–8	R
Levofloxacin	MIC	≥8	2–8	R
Ciprofloxacin	MIC	≥4	1–4	R
Ceftazidime	MIC	≥64	8–32	R
Tobramycin	MIC	≥16	4–16	R
Imipenem	MIC	≥16	2–8	R
Doxycycline	MIC	≥16	4–16	R
SXT	MIC	8/152	2–4	R
Minocycline	MIC	2	4–16	S
Cefepime	MIC	≥32	8–32	R
Piperacillin-azobactam	MIC	≥128	16–128	R
Cefoperazone-sulbactam	MIC	32	16–64	I
Colistin	MIC	≤0.5	2–4	S
Tigecycline	MIC	1	2–8	S
Ticarcillin-clavulanic	MIC	≥128	16–128	R

SXT, sulfamethoxazole-trimethoprim; MIC, minimum inhibitory concentration; R, resistant; I, intermediate; S, sensitive.

#### 2.2.2 Febrile neutropenia event

On April 18 (day +9 post-transplant), the patient developed severe neutropenia (0 × 10^9^/L) with fever, diarrhea and received supplemental oxygen at 3 L/min via nasal cannula to maintained normal oxygen saturation due to tachypnea and mild hypoxemia. These symptoms were once thought to reflect engraftment syndrome, but the patient had persistent diarrhea thereafter. Antibiotic treatment was escalated to cefoperazone-sulbactam (1:1 formulation, sulbactam 150 mg/kg/d) combined with minocycline. However, the fever persisted and teicoplanin was added to cover Gram-positive organisms on April 18, but with poor response.

#### 2.2.3 CRAB associated BSI

On April 21 (day +12 post-transplant), the patient's condition deteriorated markedly, with recurrent high fever, hypoxemia, and pulmonary crackles. Inflammatory markers including C-reactive protein and procalcitonin progressively increased. Concurrently, blood samples were sent for chimerism analysis, catheter blood culture, and metagenomic next-generation sequencing (mNGS) for confirming the infectious pathogen. Due to patient noncooperation and severe thrombocytopenia, we were unable to obtain peripheral blood specimens for microbiological culture. Chimerism analysis revealed paternal donor chimerism of only 0.63%, confirming second primary graft failure. mNGS demonstrated polymicrobial infection including Acinetobacter baumannii (read count: 1,535), Saccharomyces cerevisiae (read count: 29,549), Candida tropicalis (read count: 62), cytomegalovirus (read count: 33,923), and herpes simplex virus type 1 (read count: 2,560). Given the second primary graft failure and severe neutropenia, all detected pathogens were considered potentially pathogenic. The antimicrobial regimen was adjusted immediately. Minocycline was discontinued and replaced with tigecycline in combination with cefoperazone-sulbactam for CRAB infection. Voriconazole was discontinued, and liposomal amphotericin B, caspofungin, and foscarnet were initiated for antifungal and antiviral therapy.

#### 2.2.4 Salvage therapy and outcome

On April 26 (day +17 post-transplant), catheter blood culture confirmed CRAB associated BSI. The results of antibiotic susceptibility are present in Table 2. Due to severe thrombocytopenia posing a high bleeding risk for catheter removal, and following multidisciplinary team discussion and ethics committee approval, the antimicrobial regimen was adjusted on April 27. Cefoperazone-sulbactam and tigecycline were discontinued, and off-label use of SUL-DUR with a dose of 200 mg/kg/d (every 6 h, with an infusion duration of 6 h) combined with meropenem (20 mg/kg, every 6 h) was initiated. The patient's temperature gradually normalized, and follow-up inflammatory markers progressively declined. Catheter blood cultures persistently converted to negative. On May 9 (day +30 post-transplant), catheter blood mNGS converted to negative for all pathogens. On May 11 (day +32 post-transplant), SUL-DUR, meropenem, and liposomal amphotericin B were discontinued. Due to pulmonary inflammation, cefoperazone-sulbactam (1:1 formulation, sulbactam 150 mg/kg/d) plus tigecycline was continued for consolidation until May 25, with no subsequent active infection observed.

**Table 2:** Antibiotic susceptibility of the Acinetobacter baumannii isolate obtained from catheter blood culture.

Antibiotic	Method	Result(µg/mL)	Interpretive ranges	Interpretation
Meropenem	MIC	≥16	2–8	R
Levofloxacin	MIC	≥8	2–8	R
Ciprofloxacin	MIC	≥4	1–4	R
Ceftazidime	MIC	≥64	8–32	R
Tobramycin	MIC	≥16	4–16	R
Imipenem	MIC	≥16	2–8	R
Doxycycline	MIC	≥16	4–16	R
SXT	MIC	8/152	2–4	R
Minocycline	MIC	2	4–16	S
Cefepime	MIC	≥32	8–32	R
Piperacillin-azobactam	MIC	≥128	16–128	R
Cefoperazone-sulbactam	MIC	≥64	16–64	R
Colistin	MIC	2	2–4	S
Tigecycline	MIC	1	2–8	S
Ticarcillin-clavulanic	MIC	≥128	16–128	R
Sulbactam-durlobactam	KB	25mm	14–16 mm	S

SXT, sulfamethoxazole-trimethoprim; MIC, minimum inhibitory concentration; R, resistant; I, intermediate; S, sensitive.

**Figure 1:**
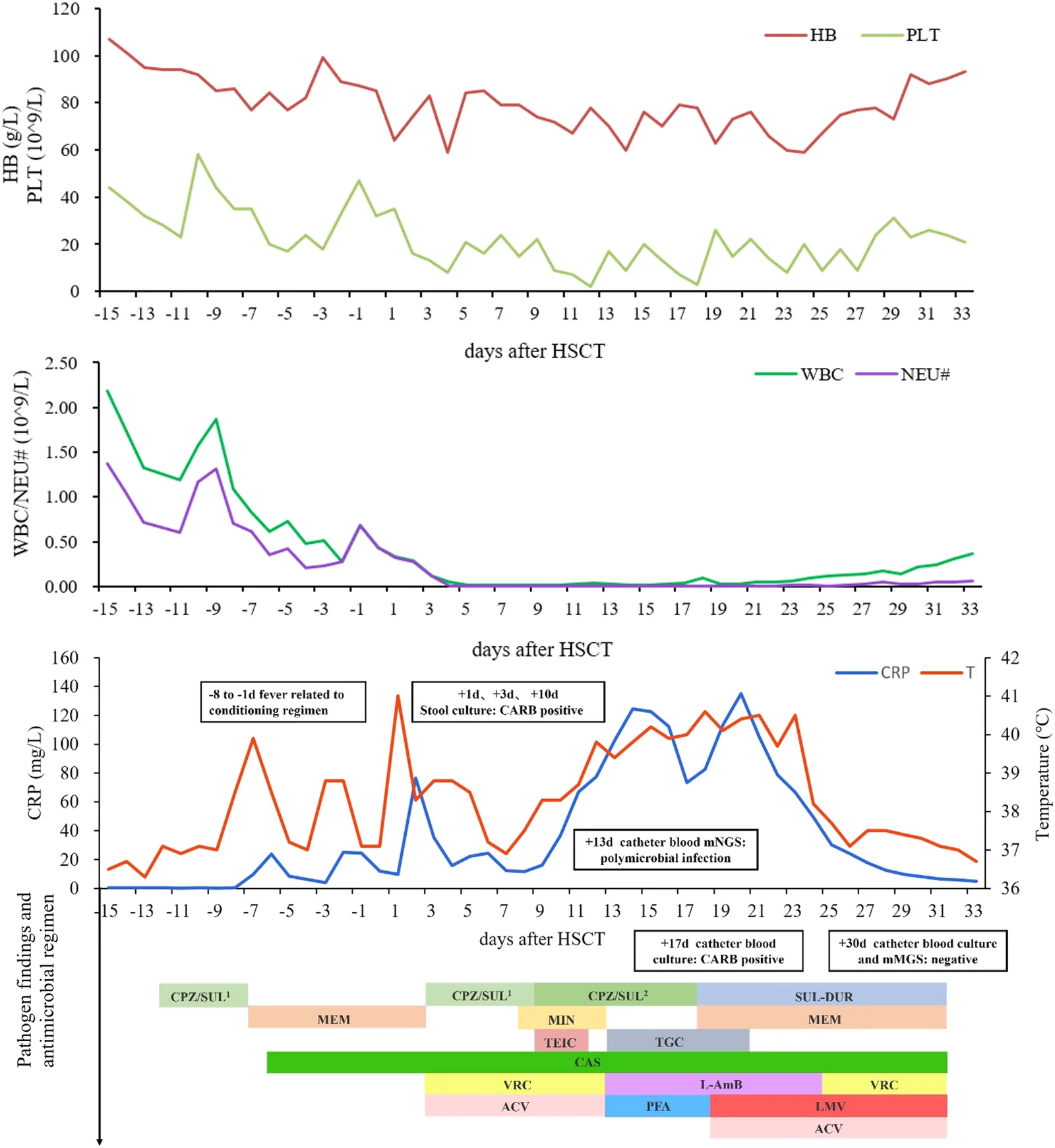
Changes in blood cell count, temperature and CRP, together with microbiological findings and antimicrobial regimen adjustments in this case. HB, hemoglobin; PTL, platelet; WBC, white blood cell; NEU#, neutrophile granulocyte; CRP, C-reactive protein; mNGS, metagenomic next-generation sequencing; CRAB, carbapenem-resistant acinetobacter baumannii; CPZ/SUL, cefoperazone-sulbactam; SUL-DUR, sulbactam-durlobactam; MEM, meropenem; MIN, minocycline; TEIC, teicoplanin; TGC, tigecycline; CAS, caspofungin; VRC, voriconazole; L-AmB, liposomal amphotericin B; ACV, acyclovir; PFA, phosphonoformic acid; LMV, letermovir.

For financial reasons, the family declined a third HSCT during the waiting period for hematopoietic recovery. No adverse drug reactions or recurrent infection events were observed during the SUL-DUR treatment period or within the following months. The temporal changes in white blood cell count, absolute neutrophil count, platelet count, hemoglobin, body temperature and C-reactive protein, together with microbiological findings and antimicrobial regimen adjustments during conditioning regimen and anti-CRAB therapy are illustrated in Figure 1.

## 3 Discussion

This article reports a case of a child with severe beta-thalassemia who experienced two failed HSCT. While in a state of persistent severe neutropenia and with a central venous catheter, the patient developed a CRAB associated BSI. After failing to respond to high-dose sulbactam (150 mg/kg/day) combined with tigecycline as anti-CRAB therapy, the infection was successfully salvaged with a regimen of SUL-DUR combined with meropenem. To our knowledge, this is the first reported case of SUL-DUR treatment for CRAB associated BSI in pediatric HSCT recipient.

BSI caused by CRAB represent a serious complication in patients undergoing HSCT, significantly associated with high mortality (15). The combined effects of transplant-related immunosuppression, prolonged neutropenia, mucosal barrier injury, and central venous catheters significantly increase the risk of BSI, as well as its persistence (16). In this case, the patient underwent routine stool carbapenem-resistant organisms colonization surveillance to reduce carbapenem exposure prophylactically. Upon development of febrile neutropenia, empirical escalation to high-dose sulbactam combined with minocycline for anti-CRAB therapy was initiated, with teicoplanin added for Gram-positive coverage (17). Concurrently, catheter blood was sent for mNGS pathogen detection and chimerism analysis. Based on mNGS detection results, antifungal and antiviral coverage was expanded, and tigecycline combined with high-dose sulbactam was used for intensified anti-CRAB therapy (18). The patient continued to experience persistent high fever with rebound of inflammatory markers after initial decline. Catheter blood culture confirmed CRAB positivity, with susceptibility testing revealing resistance to cefoperazone-sulbactam/MIC ≥64 μg/mL, while maintaining susceptibility to tigecycline, polymyxin, and minocycline with unchanged MICs. Additional susceptibility testing for SUL-DUR also demonstrated susceptibility. This phenotypic change reveals the limitations of conventional regimens in profoundly immunosuppressed hosts (19, 20).

Tigecycline exhibits poor penetration into blood and lung epithelial lining fluid, limiting its utility in bloodstream infections and pneumonia (2). Minocycline has a narrow pharmacokinetic and pharmacodynamic target attainment window. If more stringent bactericidal targets are applied, an MIC ≤0.5 mg/L is typically required (21). However, this patient's catheter blood CRAB isolate demonstrated an MIC of 4 μg/mL for minocycline, suggesting its suitability primarily as part of combination therapy for isolates with lower MIC. Polymyxins is associated with significant renal and neurotoxicity, with poor tolerability in pediatric HSCT recipients following conditioning regimens (2). As for high-dose sulbactam, limited clinical data support the efficacy of sulbactam monotherapy for CRAB infections. Current recommendations advocate for at least two antimicrobial agents in combination, and CRAB resistance to sulbactam may intensify during sustained therapy (22). This theoretical concern was indeed confirmed in this patient's infectious course, emphasizing the necessity of dynamic susceptibility monitoring. Furthermore, the absence of host clearance capacity demands that antimicrobial agents possess potent bactericidal activity, which may further diminish the therapeutic response to sulbactam (23).

Early catheter removal is recommended when CRAB catheter-related BSI is suspected (24). However, in this case, the patient had a prolonged febrile course, and concurrent internal and external catheter blood cultures were not obtained at the time of diagnosis. Following salvage therapy, both internal and external catheter blood cultures were negative at 24-h re-evaluation. We could only confirm CRAB associated BSI and could not definitively diagnose catheter-related BSI. Due to refractory thrombocytopenia posing a high bleeding risk, catheter removal was not feasible at that time. While maintaining antifungal and antiviral therapy, cefoperazone-sulbactam and tigecycline were discontinued, and SUL-DUR combined with meropenem was initiated as salvage therapy. This successfully controlled the infection, halted sepsis progression, and eliminated the persistent inflammatory stimulus from CRAB associated BSI. This regimen demonstrated satisfactory efficacy and safety.

SUL-DUR, as a novel β-lactam-β-lactamase inhibitor combination, restores the bactericidal activity of sulbactam against CRAB by inhibiting class A, C, and D serine beta-lactamases, including OXA-type carbapenemase (11). The recent IDSA guideline on the treatment of antimicrobial-resistant Gram-Negative infections recommend sulbactam-durlobactam plus meropenem or imipenem-cilastatin as the preferred therapy for CRAB infections. The addition of a carbapenem (imipenem or meropenem) enhances sulbactam-durlobactam activity through complementary PBP binding-sulbactam targets PBP1a/1b and PBP3. This combination enhances bactericidal effect through dual blockade of cell wall synthesis, potentially effective against CRAB infection. Moreover, both SUL-DUR and meropenem possess excellent tissue penetration, achieving effective concentrations in blood, respiratory tract, and soft tissues, expanding the antibacterial spectrum and proving critical for controlling CRAB infection including bloodstream infection and pneumonia (19). This case successfully demonstrates that SUL-DUR can effectively clear biofilm-associated CRAB infection in the setting of a retained catheter, providing important clinical pharmacodynamic evidence for the high-risk pediatric transplant population.

This study has several limitations. It represents a single case and limits the generalizability of conclusion. There is absence of pharmacokinetic data of SUL-DUR in pediatric and population PK studies are needed to define optimal dosing, infusion strategies and target attainment rates. Besides, the whole-genome sequencing of the CRAB isolate was not performed, precluding clarification of the association between resistance gene evolution and therapeutic pressure. Further studies in larger pediatric cohorts are needed to evaluate the role of SUL-DUR in CARB infection. Despite these limitations, the successful salvage therapy by SUL-DUR in this patient supports further investigation of this approach in pediatric HSCT recipients suffering CRAB associated BSI.

## 4 Conclusion

Our real-world evidence suggests that SUL-DUR is efficient and safe for CRAB associated BSI in pediatric patient and provides a novel therapeutic strategy for the management of this multidrug-resistant bacterial infections in high-risk pediatric transplant populations.

## Data Availability

The datasets presented in this article are not readily available because of ethical and privacy restrictions. Requests to access the datasets should be directed to the corresponding author.
